# *Polygonum* *perfoliatum* L., an Excellent Herbal Medicine Widely Used in China: A Review

**DOI:** 10.3389/fphar.2020.581266

**Published:** 2020-11-16

**Authors:** Junyu Liu, Yuanlian Zeng, Guojuan Sun, Shaopeng Yu, Yi Xu, Chao He, Zulun Li, Shenrui Jin, Xuhua Qin

**Affiliations:** ^1^ College of Pharmacy, Chengdu University of Traditional Chinese Medicine, Chengdu, China; ^2^ Hospital of Chengdu University of Traditional Chinese Medicine, Chengdu, China; ^3^ College of Basic Medicine, Chengdu University of Traditional Chinese Medicine, Chengdu, China

**Keywords:** *Polygonum perfoliatum* L., botany, traditional uses, phytochemistry, pharmacological activities, quality control

## Abstract

*Polygonum perfoliatum* L. (synonym: *Polygonum knotweed* L.; *Persicaria perfoliata*; family: Polygonaceae) is a kind of folk traditional Chinese medicine with a long history of wide use in the treatment of ancient internal, surgical, and gynecological diseases. At present, 80 chemical constituents have been isolated from *P. perfoliatum*, including flavonoids, anthraquinones, terpenoids, phenolic acids, phenylpropanoids, and alkaloids, among which flavonoids are the main active components. Modern studies have shown that *P. perfoliatum* has pharmacological activities such as anti-inflammatory, anti-bacterial, antiviral, anti-liver fibrosis, antitussive and expectorant, anti-tumor, anti-oxidation, and so on. By consulting and sorting out a large number of related literatures at home and abroad in recent years, this paper systematically reviewed the botany, traditional uses, phytochemistry, pharmacological activities, and quality control of *P. perfoliatum*, and discussed its development potential in new drug research and clinical application in the future, in order to provide a reference basis for further research and promote the in-depth development and utilization.

## Introduction

*P. perfoliatum* is a kind of folk traditional Chinese medicine, which has a long history of drug use in China, and its resources are widely distributed in many areas of Asia (Editorial Board of the State Administration of TCM, 1998; Zhao et al., 2006). Before people realized its application values, it was once regarded as a weed and classified as harmful agricultural plants for control (Gao et al., 2013); because of its strong vitality and strong ability to adapt to the environment, it can grow almost anywhere. However, *P. perfoliatum* integrates food, feeding, and medicine, which can not only be collected and processed into delicious dishes but also as a high-quality livestock and poultry feeding plants. Normal consumption and feeding are beneficial to the health of human beings and animals, and it also has high medicinal value (Ye and Du, 2014). In ancient China, the *P. perfoliatum* was initially used to treat venomous snake bites. It is said that a person bitten by a poisonous snake was carried to see a doctor on a board, and after using the *P. perfoliatum* for treatment, he was able to carry the board back home by himself. This is why, in Chinese, *P. perfoliatum* is named Kang Ban Gui, which means the person who carries the board back. Later, with the accumulation of clinical experience, it has been found that *P. perfoliatum* can also be used to treat many diseases such as sore throat, lung heat cough, infantile cough, edema and oliguria, damp-heat diarrhea, eczema, furuncles, and so on (State Pharmacopoeia Committee, 2020).

Nowadays, drugs with *P. perfoliatum* as the main raw material are widely used in the treatment of internal medicine, surgery, and gynecological diseases, and the curative effect is remarkable (Lu, 2011). At present, 80 chemical constituents have been isolated from *P. perfoliatum*, including flavonoids, anthraquinones, terpenoids, phenolic acids, phenylpropanoids, and alkaloids, showing a wide range of pharmacological activities such as anti-inflammatory, anti-bacterial, antiviral, and so on. Recently, with an increasing interest in the study of the chemical constituents of *P. perfoliatum*, domestic and overseas researchers have also achieved significant findings in liver diseases, such as hepatoprotective, anti-liver fibrosis, etc.

In this paper, with *Polygonum perfoliatum* L. as the key word, the botany, traditional uses, phytochemistry, pharmacological activities, and quality control of *P. perfoliatum* were summarized by searching the relevant literatures in China National Knowledge Infrastructure, Wang Fang, PubMed, Web of Science, and other databases, and discusses the research direction and focus in the future, so as to provide reference for further research, development, and utilization of *P. perfoliatum*.

## Botany

**Figure 1:**
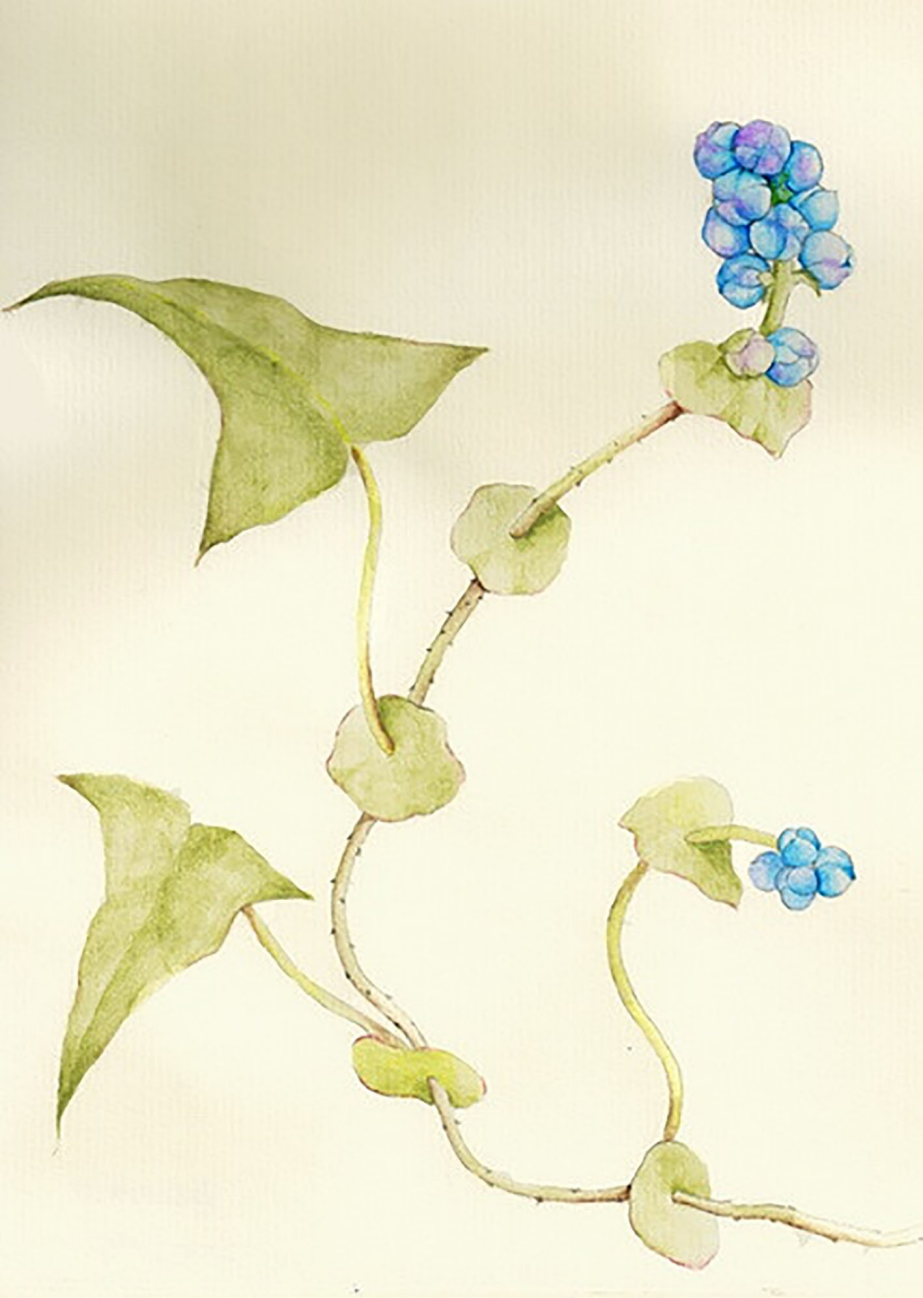
The whole plant.

*P. perfoliatum* is an annual Chinese herbal belonging to the genus Polygonum of Polygonaceae; its stem is bent, much branched, and the whole plant is approximately1‒2 meters in height. The plant is longitudinally angled, with sparse anatropous prickles along ribs. The shape of leaves is triangular, about 3‒7 cm in length and 2‒5 cm in width, its apex is obtuse or apical, and the base is truncate or puberulent, thinly papery, glabrous above, sparsely prickly below along veins. The petiole is nearly as long as leaf blade, with anatropous prickles, peltate inserted near base of leaf blade; the stipule like a sheath, herbaceous, green, with orbicular or suborbicular shape, piercing, 1.5‒3 cm in diameter. Its racemes are like short spike unbranched terminal or axillary, about 1‒3 cm in length; the bracts are ovoid, with 2‒4 flowers in each bract; it has 5 deeply clefted perianth, with white or reddish, perianth segments oval, about 3 mm long, which increase with the fruit and become fleshy and dark blue; there are 8 stamens slightly shorter than perianth; the style is trigeminal, connate in the middle and upper parts. The stigma is capitate, achene globose, 3‒4 mm in diam, black glossy, enclosed in persistent perianth. The florescence is from June to August, and fruiting period is from July to October. The whole plant and its different parts of *P. perfoliatum* are as shown in **Figure 1**. *P. perfoliatum* normally grows in valleys, bushes, fields, or ditches, and it has a strong ability to adapt to survival, but it likes a warm and sunny environment. *P. perfoliatum* has a wide range of producing areas, not only in China but also in North Korea, Japan, Indonesia, the Philippines, India, Russia (Siberia), and other places (Zhang, 1998; Tan et al., 2013).

## Traditional Uses

As a folk traditional Chinese medicine with a long history of use, *P. perfoliatum* has a variety of effects of food, feeding, and medicine (Ye and Du, 2014), and it is often used as raw food in its tender leaves in the corners of the fields, or its tender stems and leaves are used as dishes; its stems and leaves are also used as feed for pigs, rabbits, geese, and other livestock and poultry; in some rural areas of Guizhou, sun-dried *P. perfoliatum* is mixed with corn and coarse grains to be used as cheap feed. Some people collect stems and leaves for simple medicinal purposes such as heat-clearing and detoxification, diuresis and detumescence, cough, and so on, and it is widely used in the treatment of sore throat, malaria, lung-heat cough, edema, eczema, diarrhea, hemorrhoids, boils, snakebite, and other diseases (Yang and Yun, 2012).

*P. perfoliatum* was first recorded in the classic medical book “Wang Bing Hui Chun” in the Ming Dynasty: “To treat snakebites, it is appropriate to use *P. perfoliatum*, tamping juice, mixing with alcohol, and rubbing the wound with dregs.” Essentials of Medicinal Properties of Raw Herbs (He, 2009) records that it tastes a little bitter, the medicine property is mild, the color of fruit is blue, and it is edible. Textual Research on The Name and Reality Map of Plants (Wu, 1963) records that *P. perfoliatum* has the function of promoting blood flow and treating gonorrhea. In addition, *P. perfoliatum* is listed in the quality standard of traditional Chinese medicine in Guizhou Province (Guizhou Medical Products Administration, 2003), and also recorded in many local medical books. According to the Collection of Folk Prescriptions in Guizhou (Yang and Yang, 1978), *P. perfoliatum* mixed with borneol and sesame oil can be used for the treatment of infectious pustulosis. *P. perfoliatum* is known to have the ability of diuresis and detumescence, as well as treat joint swelling and pain of the lower extremities (Fujian Medical Research Institute, 1970). Jiangxi Herbal Medicine (Jiangxi Provincial Health Bureau Revolutionary Committee, 1970) recorded that *P. perfoliatum* stewed with pig large intestine can be used to treat hemorrhoids. *P. perfoliatum* is also widely used in the treatment of herpes zoster. In ancient times, people called this viral infectious skin disease “waist wrapping fire.” It was named because it often appeared in clusters of erythema blisters on the skin of the waist, showing a banded distribution, and its hurts like being roasted by fire. People collected the *P. perfoliatum* from the valleys, fields, or ditches, then mashed it and applied it to the affected area, which could effectively relieve the pain of the patient and could be cured in 3‒5 days.

Whole plant of *P. perfoliatum* can be used as medicine, which has high medicinal value, not only to be eaten but also has a certain health care effect. In the Guangxi Zhuang Autonomous region, people use *P. perfoliatum* together with other materials to make a tea drink for the treatment of hemorrhoids; its formula has now been patented (Meng, 2017). In addition, *P. perfoliatum* is also used as the raw material of a health care capsule, which is effective in treating cough and indigestion, strengthening the body, improving immunity, and moisturizing skin tone (Ni, 2018).

## Phytochemistry

Although *P. perfoliatum* has a long history of application, it started relatively late in the study of its chemical constituents. So far, more than 80 compounds have been isolated and identified from *P. perfoliatum*, including flavonoids (1‒26), anthraquinones (27‒30), terpenoids (31‒39), phenolic acids (40‒51), phenylpropanoids (52‒64), alkaloids (65‒67), and other compounds (68‒80); among them, flavonoids are the most important active components of *P. perfoliatum*.

### Flavonoids

Flavonoids are widely distributed in nature, with anti-tumor, anti-inflammatory, bacteriostatic, liver protection, antioxidant, and other physiological activities (Zhu et al., 2016), and it’s also a kind of chemical constituent that has been widely reported in *P. perfoliatum* related literature. At present, 26 flavonoids (**1‒26**) have been isolated from *P. perfoliatum*, including 13 flavonols (**1‒13**), Quercetin (**1**), Quercetin-3-O-β-D-glucoside (**2**), Quercetin-3-O-β-D-glucuronide-6″-butyl ester (**3**), Quercetin-3-O-β-D-glucuronide-6″-methyl ester (**4**), Quercetin-3-O-β-D-glucuronide (**5**), Quercetin-4’-O-β-D-glucuronide (**6**), Quercitrin (**7**), Isorhamnetin (**8**), Hyperoside (**9**), Kaempferol (**10**), Kaempferol-3-O-rutinoside (**11**), Rutin (**12**), and Avicularin (**13**).

**Figure 2:**
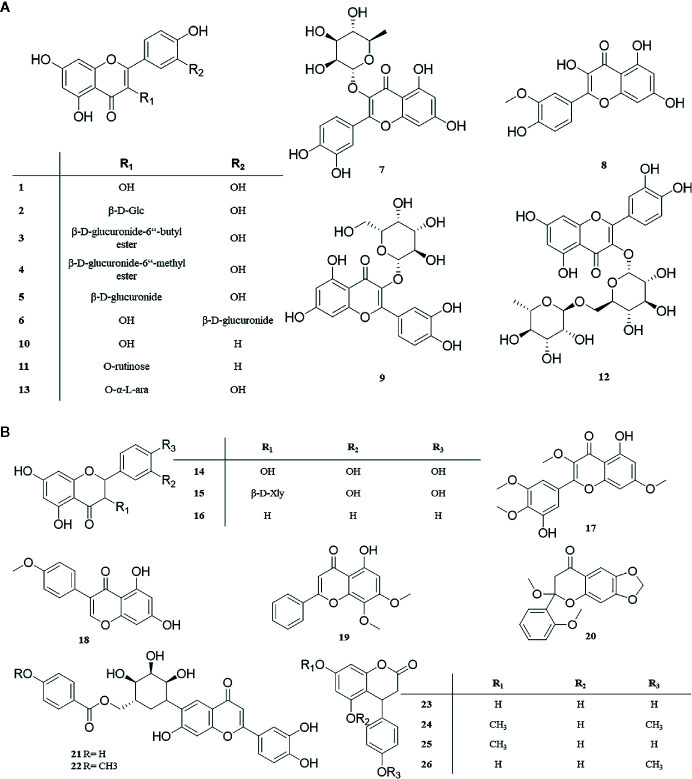
**(A, B)** Structure of flavonoids from *P. perfoliatum*.

Cheng et al. (2012) isolated three dihydroflavonols (**14‒16**) from the ethyl acetate extract of *P. perfoliatum*: Taxifolin (**14**), Taxifolin-3-O-β-D-xylopyranoside (**15**), and Pinocernbrin (**16**). Six flavonoids (**17‒22**) were discovered from *P. perfoliatum*: 3’,5-dihydroxy-3,4’,5’,7-tetramathoxy-flavone (**17**), 5, 7-dihydroxy-4-methoxy isoflavone (**18**), 5-hydroxy-7,8-dimethoxy flavonoids (**19**), 2-dimethoxy-6-dimethoxy-7-methylenedioxy flavonoids (**20**), Perfoliatumin A (**21**), and Perfoliatumin B (**22**). Four new flavonoids (**23‒26**) were reported for the first time (Sun and Sneden, 1999): 3,4-dihydro-4-(4’-hydroxyphenyl)-5,7-dihydroxy- coumarin (**23**), 3,4-dihydro-5-hydroxy-7-methoxy-4-(4’-methoxyphenyl) coumarin (**24**), 3,4-dihydro-5-hydroxy-4-(4’-hydroxyphenyl)-7-methoxycoumarin (**25**), and 3,4-dihydro-5,7-dihydroxy-4-(4’-methoxyphenyl) coumarin (**26**). These compounds and their corresponding structures are shown in **Table 1** and **Figures 2A, B**.

**Table 1:** Chemical constituents of flavonoids from *Polygonum perfoliatum* L.

Classification	No.	Name	Reference
Flavonols	1	Quercetin	Wang and Lu, 2004
	2	Quercetin-3-O-β-D-glucoside	Wang and Lu, 2004
	3	Quercetin-3-O-β-D-glucuronide-6″-butyl ester	Zhang et al., 2008
	4	Quercetin-3-O-β-D-glucuronide-6″-methyl ester	Pan et al., 2017
	5	Quercetin-3-O-β-D-glucuronide	Pan et al., 2017
	6	Quercetin-4’-O-β-D-glucuronide	Xu, 2010
	7	Quercitrin	Wang et al., 2009
	8	Isorhamnetin	Wang et al., 2009
	9	Hyperoside	Wang et al., 2009
	10	Kaempferol	Zhang et al., 2008
	11	Kaempferol-3-O-rutinoside	Zhang et al., 2008
	12	Rutin	Zhang et al., 2008
	13	Avicularin	Zhang et al., 2008
Dihydroflavonols	14	Taxifolin	Cheng et al., 2012
	15	Taxifolin-3-O-β-D-xylopyranoside	Cheng et al., 2012
	16	Pinocernbrin	Cheng, 2012
Flavonoids	17	3’,5-dihydroxy-3,4’,5’,7-tetramathoxy-flavone	Wang et al., 2012
	18 19	5, 7-dihydroxy-4-methoxy isoflavone 5-hydroxy-7,8-dimethoxy flavonoids	Zhu et al., 2000 Zhu et al., 2000
	20	2-dimethoxy-6-dimethoxy-7-methylenedioxy flavonoids	Zhu et al., 2000
	21	Perfoliatumin A	Zhu et al., 2000
	22	Perfoliatumin B	Zhu et al., 2000
New Flavonoids	23	3,4-dihydro-4-(4’-hydroxyphenyl)-5,7-dihydroxy- coumarin	Sun and Sneden, 1999
	24	3,4-dihydro-5-hydroxy-7-methoxy-4-(4’-methoxyphenyl) coumarin	Sun and Sneden, 1999
	25	3,4-dihydro-5-hydroxy-4-(4’-hydroxyphenyl)-7-methoxycoumarin	Sun and Sneden, 1999
	26	3,4-dihydro-5,7-dihydroxy-4-(4’-methoxyphenyl) coumarin	Sun and Sneden, 1999
Anthraquinones	27	Emodin	Wang and Lu, 2004
	28	Emodin-3-methyl ether	Wang and Lu, 2004
	29	aloe-Emodin	Wang and Lu, 2004
	30	α-tocopheroiquinone	Li et al., 2009
Tetracyclic triterpenoids	31	β-sitosterol	Wang and Lu, 2004
	32	Sitostenone	Li et al., 2009
	33	Daucosterol	Zhao et al., 2009b
	34	cucurbitacin IIa	Li et al., 2009
	35	cucurbitacin U	Li et al., 2009
	36	(24S’)-24-ethylcholesta-3β,5α,6α-triol	Li et al., 2009
Pentacyclic triterpenoids	37	Friedelin	Li et al., 2009
	38	Aster-yunnanoside F	Li et al., 2009
	39	Saikosaponin M	Li et al., 2009
Phenolic acids	40	Protocatechuic acid	Wang et al., 2009
	41	Gallic acid	Wang et al., 2009
	42	Ellagic acid	Wang et al., 2009
	43	3,3’-dimethoxy Ellagic acid	Wang et al., 2009
	44	1-O-galloyl-β-D-glucose	Wang et al., 2009
	45	Mucic acid dimethyl ester-2-O-gallate	Wang et al., 2009
	46	Caffeic acid	Zhao et al., 2009b
	47	Methyl 3,4-dihydroxycinnamate	Cheng et al., 2012
	48	Caffeic acid ethyl ester	Wang et al., 2009
	49	4-hydroxy-3-methoxycinnamic acid methyl ester	Zhao et al., 2009b
	50	Methyl 4-hydroxycinnamate	Wang and Lu, 2004
	51	4-dihydroxy-5,7-dihydroxy-4-(4-hydroxyphenyl) coumarin	Li et al., 2009
Phenylpropionic acid	52	6’-acetyl-3,6-diferuloylsucrose	Sun et al., 2000
	53	2’,4’,6’-triacetyl-3,6-diferuloylsucrose	Sun et al., 2000
	54	1, 2’,4’,6’-tetraacetyl-3,6-diferuloylsucrose	Sun et al., 2000
	55	1,2’,6’-triacetyl-3, 6-diferuloylsucrose	Sun et al., 2000
	56	2’,6’-diacetyl-3,6-diferuloylsucrose	Sun et al., 2000
Lignins	57	Matairesinol	Li et al., 2009
	58	8’-oxo’-pinoresinol	Wang et al., 2012
Coumarins	59	Esculetin	Zhao et al., 2010
	60	Skimmin	Zhao et al., 2009b
Phenylpropanoid analogues	61	Vanicoside B	Li et al., 2009
	62	Vanicoside C	Li et al., 2009
	63	Vanicoside F	Li et al., 2009
	64	Hydropiperoside	Li et al., 2009
Amide alkaloids	65	iotroridoside A	Li et al., 2009
	66	pokeweedcerebroside 5	Li et al., 2009
	67	bonaroside	Li et al., 2009
Volatile compounds	68	2-hexenal	Zhao et al., 2009a
	69	2-undecanone	Zhao et al., 2009a
	70	3-methyl-cyclopentene	Zhao et al., 2009a
	71	benzaldehyde	Zhao et al., 2009a
	72	Palmitic acid	Zhao et al., 2009a
	73	Decanoic acid	Zhao et al., 2009a
Small molecular sugars and derivatives	74	n-butyl-β-D-fructopyronoside	Zhang, 2007
	75	5-Hydroxymethyl-2-furaldehyde	Zhao et al., 2010
	76	Fructose	Zhang, 2007
Straight-chain hydrocarbons	77	Pentacosanoic acid	Xu, 2010
	78	Pentadecanoic acid	Zhang, 2007
	79	Octacosane	Zhao et al., 2009b
	80	Triacontane	Zhao et al., 2009b

### Anthraquinones

**Figure 3:**
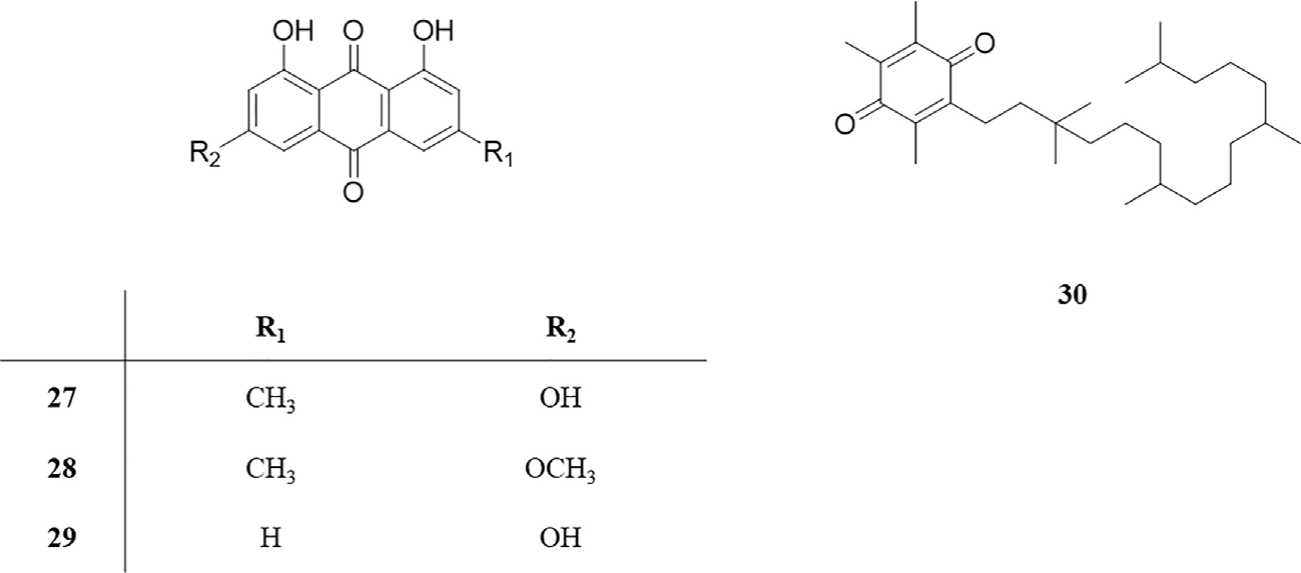
Structure of anthraquinones from *P. perfoliatum*.

Anthraquinones are abundant in polygonaceae plants, which have many pharmacological effects, such as anti-virus, anti-tumor, liver protection, blood lipid lowering, blood pressure lowering, antibacterial, anti-inflammatory, and so on. Four anthraquinones, emodin (**27**), emodin-3-methyl ether (**28**), aloe-emodin (**29**), and α-tocopheroIquinone (**30**), were isolated from the roots of *P. perfoliatum* for the first time in 2004 (Wang and Lu, 2004); after that, no new compounds were reported. These anthraquinones and their corresponding structures are shown in **Table 1** and **Figure 3**.

### Terpenoids

**Figure 4:**
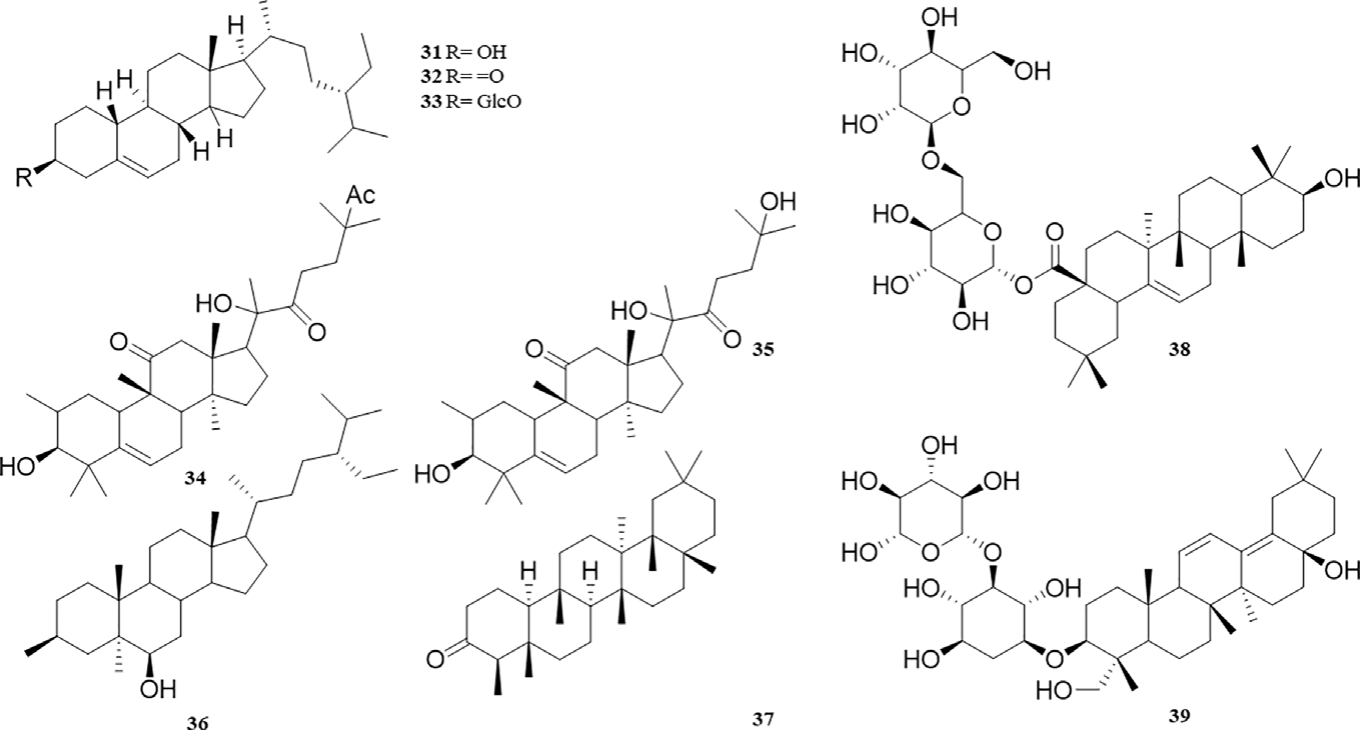
Structure of Terpenoids from *P. perfoliatum*.

Terpenoids in *P. perfoliatum* are rarely reported. At present, 9 terpenoids have been isolated from *P. perfoliatum*, including 6 tetracyclic triterpenoids with basic structure of cyclopentano polyhydrophenanthrene (**31‒36**), β-sitosterol (**31**), Sitostenone (**32**), Daucosterol (**33**), cucurbitacin IIa (**34**), cucurbitacin U (**35**), and (24S’)-24-ethylcholesta-3β,5α,6α-triol (**36**). The other 3 compounds are pentacyclic triterpenoids (**37‒39**), including Friedelin (**37**), Aster-yunnanoside F (**38**), and Saikosaponin M (**39**). These compounds and their corresponding structures are shown in **Table 1** and **Figure 4**.

### Phenolic Acids

**Figure 5:**
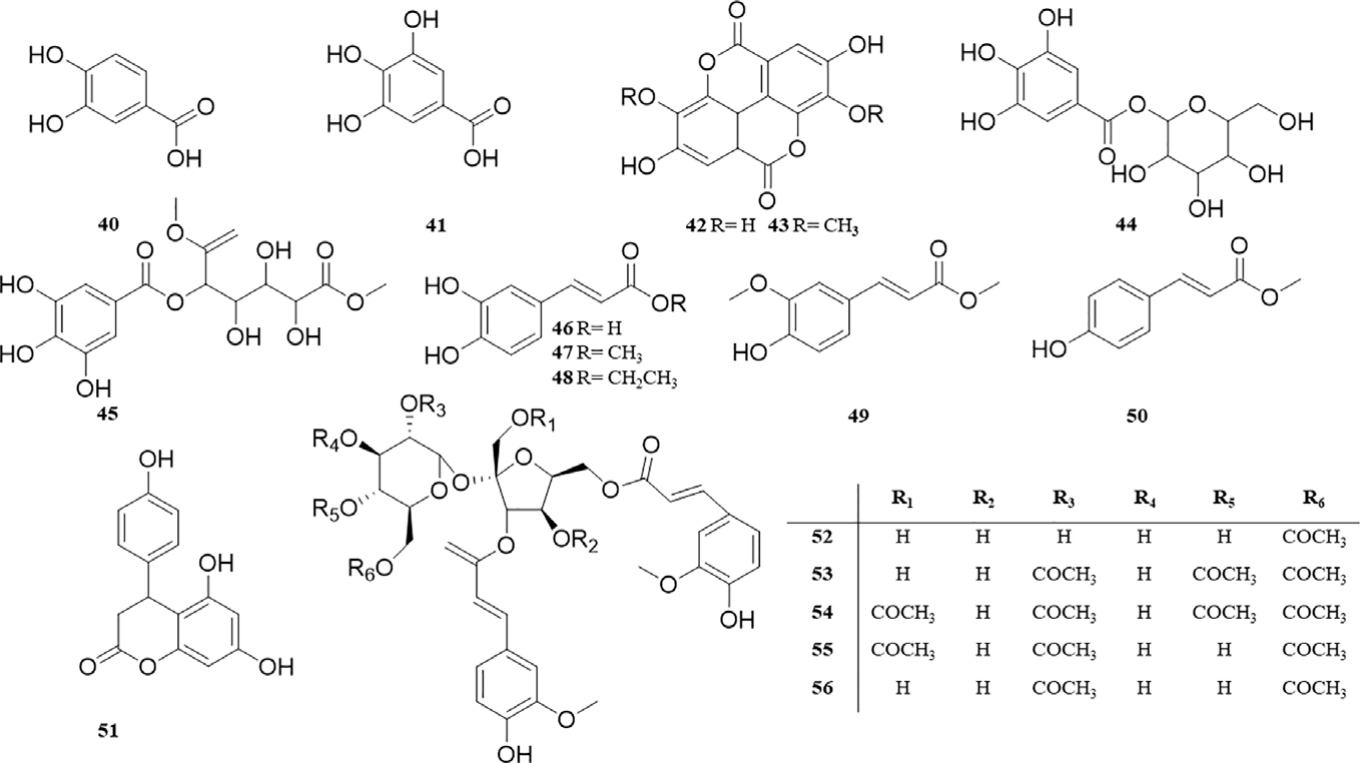
Structure of phenolic acids from *P. perfoliatum*.

The chemical constituents of phenolic acids in *P. perfoliatum* are polar, and the active components are mainly enriched in n-butanol and water extracts. It has been found that phenolic acids have anti-inflammatory and antiviral activities, which may be related to the Gallic acyl groups in the structure. At present, 12 compounds of phenolic acids have been isolated from *P. perfoliatum* (**40‒51**). Wang et al. (2009) reported 7 compounds: Protocatechuic acid (**40**), Gallic acid (**41**), Ellagic acid (**42**), 3,3’-dimethoxy Ellagic acid (**43**), 1-O-galloyl-β-D-glucose (**44**), Mucic acid dimethyl ester-2-O-gallate (**45**), and Caffeic acid ethyl ester (**48**). The other 5 compounds are Caffeic acid (**46**), Methyl 3,4-dihydroxycinnamate (**47**), 4-hydroxy-3-methoxycinnamic acid methyl ester (**49**), Methyl 4-hydroxycinnamate (**50**), and 4-dihydroxy-5,7-dihydroxy-4-(4-hydroxyphenyl) coumarin (**51**).These compounds and their corresponding structures are shown in **Table 1** and **Figure 5**.

### Phenylpropanoids

**Figure 6:**
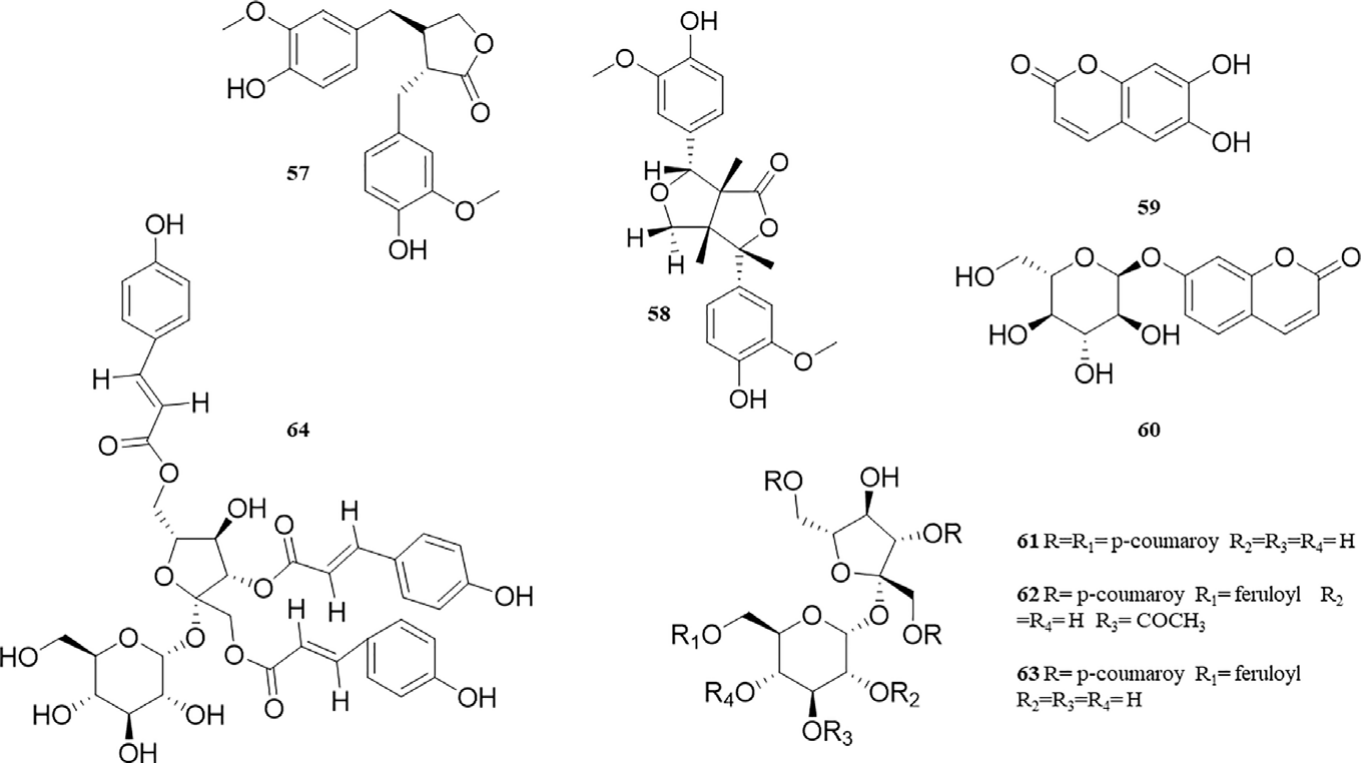
Structure of phenylpropanoids from *P. perfoliatum*.

Thirteen phenylpropanoids (**52‒64**) were proven to exist in *P. perfoliatum*. Five phenylpropionic acid compounds include 6’-acetyl-3,6-diferuloylsucrose (**52**), 2’,4’,6’-triacetyl-3,6-diferuloylsucrose (**53**), 1,2’,4’,6’-tetraacetyl-3,6-diferuloylsucrose (**54**), 1,2’,6’-triacetyl-3, 6-diferuloylsucrose (**55**), and 2’,6’-diacetyl-3,6-diferuloylsucrose (**56**). There are 2 lignins isolated from *P. perfoliatum*: 7,7’-dihydroxymatairesinol (**57**) and 8’-oxo’-pinoresinol (**58**). Esculetin (**59**) and Skimmin (**60**), two coumarins, were separated from *P. perfoliatum*. Li et al. (2009) separated 4 phenylpropanoid analogues: Vanicoside B (**61**), Vanicoside C (**62**), Vanicoside F (**63**), and Hydropiperoside (**64**). These compounds and their corresponding structures are shown in **Table 1** and **Figure 6**.

### Alkaloids and Other Compounds

**Figure 7:**
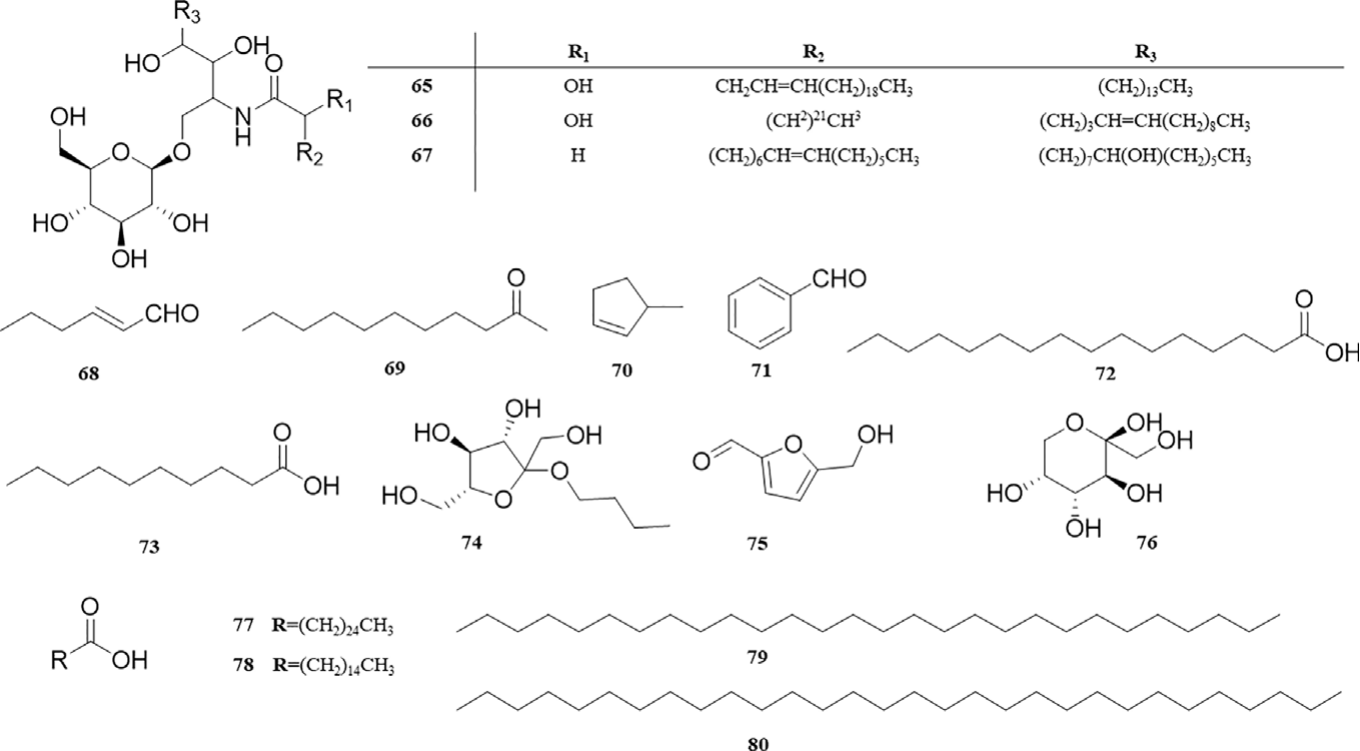
Structure of alkaloids and other compounds from *P. perfoliatum*.

Amide alkaloids (**65‒67**), volatile components (**68‒73**), and other components (**74‒80**) were also isolated from *P. perfoliatum*. Amide alkaloids, such as IotroridosideA (**65**), pokeweedcerebroside 5 (**66**), and bonaroside (**67**), were isolated from *P. perfoliatum* for the first time (Li et al., 2009). Zhao et al. (2009a) obtained volatile components such as 2-hexenal (**68**), 2-undecanone (**69**), 3-methyl-cyclopentene (**70**), benzaldehyde (**71**), palmitic acid (**72**), and decanoic acid (**73**) from *P. perfoliatum* by solid phase microextraction (SPME). In addition, *P. perfoliatum* also contains some small molecular sugars and straight-chain hydrocarbons: n-butyl-β-D-fructopyronoside (**74**), 5-Hydroxymethyl-2-furaldehyde (**75**), Fructose (**76**), Pentacosanoic acid (**77**), Pentadecanoic acid (**78**), Octacosane (**79**), and Triacontane (**80**). These compounds and their corresponding structures are shown in **Table 1** and **Figure 7**.

## Pharmacological Activities

Modern pharmaceutical studies have shown that different extracts of *P. perfoliatum* have anti-inflammatory, bacteriostatic, anti-viral, anti-liver fibrosis, antitussive and expectorant, anti-tumor, and other pharmacological activities (**Table 2**). Clinically, it is widely used in the treatment of a variety of viral infectious skin diseases and gynecological inflammatory diseases, such as herpes zoster, eczema, and pelvic inflammation. However, most of the published studies lack information about the identification of their active compounds, their action mechanisms and targets in vivo, and related extract preparations, which need to be further explored and studied.

**Table 2:** Pharmacological activities of *P. perfoliatum*.

Pharmacological activities	Experimental object/Method	Part of plant/Extracts	Dose/Duration	Control		Results	Reference
				Positive	Negative		
Anti-inflammation	Kunming mice/*In vivo*	The dried aboveground parts/Ethanol, ethyl acetate, n-butanol	78 mg/20 g/7 d	Aspirin	Saline	The ethanol extract and n-butanol extract of the plant has anti-inflammatory activity, and the ethyl acetate extract does not show anti-inflammatory activity.	Huang et al., 2008
	Kunming mice, Wistar rats/*In vivo*	The dried aboveground parts/Ethanol	5 g/kg/1 d/5 d	Aspirin	Saline	The ethanol extract of the plant can reduce the contents of PGE2 and MDA in serum. It has an inhibitory effect on early inflammation.	Long and Li, 2010
	Wistar rats/*In vivo*	GBG	0.27, 0.54, 0.81 g/kg/14 d	FuYanKang	Saline	GBG can significantly reduce uterine inflammation and has a good therapeutic effect on pelvic inflammation.	Zhang Q. et al., 2012
	Wistar rats/*In vivo*	GBG	0.27, 0.54, 0.81 g/kg/5 d	Aspirin	Saline	GBG can significantly inhibit foot swelling and granulation tissue formation of cotton balls in rats, and has strong anti-inflammatory effect.	Zhang et al., 2013
	Kunming mice/*In vivo*	Whole plant/petroleum ether	50, 100 mg/mL	Compound dexamethasone acetate cream	NA	The petroleum ether extract of the plant has the strongest anti-inflammatory activity, and the swelling inhibition rate is 71.60%.	Fan et al., 2011
Anti-bacterial	S. aureus, Kober-6, E. coli, SS II-6, Amur72/*In vitro*	Dried whole plant/Distilled water, methanol, ethanol	1~0.008 g/mL/24 h	NA	Blank medium	The water extract of plant can effectively inhibit S. aureus, Kober-6, E. coli, SS II-6, Amur72, and other strains.	Fu et al., 2008
	S. aureus, E. coli, Proteus, P. aeruginosa and B. subtilis/*In vitro*	Dried whole plant/Ethanol, petroleum ether, ethyl acetate, n-butanol	1.25 ×10^-2^ ~ 80×10^-2^ mg/mL/24 h	Streptomycin	Saline	The ethanol extract of the plant has strong inhibitory effect on S. aureus, Proteus, P. aeruginosa, and B. subtilis, and its MIC was 5.0 × 10^-2^, 10.0 × 10 ^-2^, 10.0 × 10 ^-2^, and 5.0 × 10^-2^ mg/mL, respectively.	Huang et al., 2013
Anti-virus	Herpes simplex virus-1/*In vitro*	Dried whole plant/Ethanol, macroporous resin	2, 4, 8, 16 μg/mL/NA	Acyclovir	Blank medium	The ethanol extract and macroporous resin ethanol elution part have significant antiviral effect (8 μg/mL).	Zhang et al., 2010
	Influenza A virus/*In vivo*	NA/Quercetin-3-O-β-D-glucuronide	3, 6 mg/kg/4 d	Ribavirin	Distilled water	Quercetin-3-O-β-D-glucuronide had a significant inhibitory effect on pulmonary edema induced by influenza A virus in mice (6mg/kg).	Fan et al., 2011
	Hepatitis B virus/*In vitro*	Dried whole plant/ethanol, n-butanol	6.25~200 µg/mL/8d	NA	Blank medium	The ethanol extract, n-butanol extract, and water extract could effectively inhibit the secretion of Hepatitis B virus, in a concentration-dependent manner.	Wang, 2009
Hepatoprotection	Balb/c mice/*In vivo*	Dried whole plant/Ethanol, distilled water	10, 20 mg/kg/7 d	Biphenyl diester	Saline	The plant has the effect of protecting liver and lowering enzymes, and can reduce liver inflammation.	Wang, 2009
	Kunming mice/*In vivo*	Dried whole plant/Total flavonoid extract	0.3, 0.6 1.2 g/kg/7 d	Biphenyl diester	CMC-Na	The total flavonoid extract has a certain effect on cholestatic liver injury induced by ANIT in mice.	Cheng et al., 2018
	Kunming mice/*In vivo*	Dried whole plant/Total flavonoid extract	150, 300, 600 mg/kg/30 d	Glucuronolactone	Distilled water	The total flavonoid extract has protective effects on liver injury induced by isoniazid and rifampicin in mice and can inhibit inflammatory reaction.	Xi et al., 2017
	Kunming mice/*In vivo*	Dried whole plant/Total flavonoid extract	60, 120 mg/kg/14d	NA	Peanut oil	The total flavonoid extract has a protective effect on CCl_4_-induced liver injury in mice and can improve the immune function of spleen.	Xu et al., 2019
	Kunming mice/*In vivo*	Dried whole plant/Ethanol	0.32, 0.64, 1.28 g/kg/21 d	Biphenyl diester	Saline	The ethanol extract has a protective effect on chemical liver injury in mice, and can reduce the pathological injury of heart and kidney cells.	He et al., 2019b
	Mice/*In vivo*	Dried whole plant/Total flavonoid extract	200, 400, 600, mg/kg/14 d	Biphenyl diester	Distilled water	The total flavonoid extract has a protective effect on acute alcoholic liver injury in mice, and can resist lipid peroxidation and inhibit liver inflammation.	Wei et al., 2016
Anti-hepatic fibrosis	SD rats/*In vivo*	Dried whole plant/Ethanol	2.5, 5, 7.5 g/kg/28 d	Colchicine	Saline	The ethanol extract can antagonize DMN-induced hepatic fibrosis in rats, and its effect is related to the dosage.	Cao et al., 2012
	SD rats/*In vivo*	Dried whole plant/Ethanol	2.5, 5, 7.5 g/kg/28 d	Colchicine	Saline	The plant has a comprehensive anti-fibrotic effect, and inhibiting the expression of TGF- β1 may be one of the molecular mechanisms of its anti-hepatic fibrosis.	Cao, 2012
	SD rats/*In vivo*	Dried whole plant/Ethanol	2.5, 5, 7.5 g/kg/28 d	Colchicine	Saline	It can protect hepatocytes by reducing the content of serum transaminase AST, ALT, and has a good effect on DMN-induced liver fibrosis in rats.	Guo and Shan, 2012
	SD rats/*In vivo*	Dried whole plant/Total flavonoid extract	0.05, 0.10, 0.15 g/kg/56 d	Colchicine	Saline	Total flavonoid extract can effectively antagonize DMN-induced hepatic fibrosis in rats, which may be achieved by reducing the expression of TGF- β1 in liver tissue.	Gao et al., 2015
	SD rats/*In vivo*	Dried whole plant/Total flavonoid extract	50, 100, 200 mg/kg/56 d	Colchicine	Saline	The total flavonoid extract can effectively antagonize DMN-induced hepatic fibrosis in rats, inhibit the inflammatory reaction, and improve the pathological changes of liver.	Cao et al., 2017
	SD rats/*In vivo*	Dried whole plant/Ethanol	2.5, 5, 7.5 g/kg/28 d	Colchicine	Saline	The ethanol extract has a significant anti-hepatic fibrosis effect, and can regulate extracellular matrix metabolism by reducing the expression of HIF-1 α and VEGF.	Cao et al., 2011
	SD rats/*In vivo*	Dried whole plant/Ethanol	2.5, 5, 7.5 g/kg/42 d	Colchicine	Saline	The alcohol extract has a significant protective effect on hepatic fibrosis in rats, and its mechanism may be related to the regulation of TGF- β1/Notch signal pathway.	Gao et al., 2017
Antitussive and expectorant	ICR mice/*In vivo*	Dried whole plant/Water	0.1, 0.2 g/10g/NA	Phosphate codeine	Saline	The antitussive effect of water extract is significant and with a dose-dependent manner.	Gu, 1996
** **	ICR mice, Wistar rats/*In vivo*	Dried whole plant/Ethanol	5 g/kg/7 d	GuiLong KeChuanNing	Saline	The ethanol extract can increase the sputum excretion of rats and has a good expectorant effect.	Long and Li, 2010
Anti-tumor	ICR mice/*In vitro*	Dried whole plant/Ethanol, petroleum ether	18.75~300 ug/mL/72 h	CTX	NA	The extracts had a good inhibitory effect on a variety of tumor cells; for the same tumor cells, the inhibitory effect of ethyl acetate extract is the highest.	Tao and Zhang, 2013
	ICR mice/*In vivo*	Dried whole plant/Ethyl acetate	50, 100, 200 mg/kg/10 d	CTX	NA	The ethyl acetate extract has a good inhibitory effect on transplanted Smur180 sarcoma in mice.	Tao and Zhang, 2013
	ICR mice/*In vivo and vitro*	Dried whole plant/Ethyl acetate	3.5, 7, 14 mg/kg/8 d	CTX	NA	The ethyl acetate extract had broad-spectrum anti-tumor activity (100–120 μg/mL), and there was no toxicity in vivo and in vitro.	Li et al., 2019
Anti-oxidation	ABTS, DPPH, α-glucosidase/*In vitro*	Dried whole plant/Ethyl acetate, methanol	20~120 µg/mL/NA	NA	NA	Ethyl acetate extract and methanol extract have certain antioxidant activity, among which methanol extract has the strongest activity.	Xing et al., 2011
	DPPH, NO_2_-/*In vitro*	Dried whole plant/polyphenol extract	30~150 mg/L/NA	Vitamin C	NA	Polyphenol extract has strong antioxidant activity, and the scavenging effect is related to the mass concentration.	Cheng et al., 2020

### Anti-Inflammatory

It was found that the ethanol extract had a significant inhibitory effect on xylene-induced auricle swelling in mice, with an inhibition rate of 81.67%, while that of aspirin, a positive control drug, was only 61.83%. The swelling inhibition rates of ethyl acetate and n-butanol isolated part from the *P. perfoliatum* ethanol extract on mouse auricle were 22.39% and 41.47%, respectively, suggesting that the anti-inflammatory active components were lost or not effectively separated in the separation process of ethanol extract, and the separation and extraction process needs to be further optimized (Huang et al., 2008). The Long (Long and Li, 2010) study found that the ethanol extract could significantly inhibit the increase of abdominal capillary permeability induced by acetic acid in mice, and reduce the absorbance (OD value) of mouse peritoneal saline washing solution at 590 nm. In addition, ethanol extract could significantly reduce the contents of PGE_2_ and MDA in serum and inflammatory tissue of rats with toe swelling induced by carrageenan. It is suggested that the anti-inflammatory mechanism of *P. perfoliatum* may be related to the decrease of the content of PGE_2_ in inflammatory tissue and the inhibition of lipid peroxidation.

In the study of Zhang Q. et al. (2012), it was found that a drug with *P. perfoliatum* as the main component, named GBG, was effective in the treatment of pelvic inflammation in rats caused by plastic tube insertion into the uterus, reducing endometrial congestion and edema, reducing the rate of uterine swelling, and improving pathological damage such as uterine adhesion occlusion, epithelial cell degeneration, and necrosis and inflammatory cell infiltration. In addition, GBG can also significantly inhibit the local tissue swelling of mice caused by a variety of inflammatory substances and the formation of granulation tissue in rats, and reduce the inflammatory response in a dose-dependent manner (Zhang et al., 2013).

In the HPLC chromatogram obtained from anti-inflammatory extracts of *P. perfoliatum*, the researcher found that the peak of quercetin-3-O-β-D-glucuronide is the highest. Moreover, it was found that quercetin-3-O-β-D-glucuronide could inhibit lipid peroxidation (Shirai et al., 2001; Begum and Terao, 2002), angiotensin II-stimulated JNK activation (Kyaw et al., 2004), and the production of reactive oxygen species (ROS) (Shirai et al., 2006). The content of quercetin-3-O-β-D-glucuronide was 0.90%, which was higher than that of quercetin in *P. perfoliatum*, indicating that the quercetin-3-O-β-D-glucuronide was one of the major active compounds of *P. perfoliatum*, and it is closely related to the anti-inflammatory effect.

### Anti-Bacterial

The water extract of *P. perfoliatum* has certain inhibitory effect on Staphylococcus aureus, Pasteurella multocida, Streptococcus, Salmonella, and Escherichia coli in vitro, and the minimum inhibitory concentration (MIC) is 0.031 g/mL, 0.25 g/mL,0.063 g/mL, 0.125 g/mL, and 0.063 g/mL, respectively (Fu et al., 2008). After further study, it was found that the bacteriostatic effect of *P. perfoliatum* organic solvent extract was better than that of water extract; through the study on the antibacterial activity of petroleum ether extract, ethyl acetate extract, and n-butanol extract of the *P. perfoliatum* ethanol extract, it was found that ethyl acetate had the strongest antibacterial activity and had significant bacteriostatic effect on Staphylococcus aureus, Escherichia coli, Streptococcus faecalis, and other pathogens (Huang et al., 2008). N-butanol extract can inhibit Bacillus subtilis and Pseudomonas aeruginosa, and while petroleum ether has no inhibitory effect on the above bacteria (Huang et al., 2013), this may be related to the fact that most of the petroleum ether extracts contain chlorophyll, tannins, and other components, but most of these components have no pharmacological activity. In addition, from the prominent anti-bacterial activity of ethyl acetate and n-butanol extract, it can be inferred that the anti-bacterial components of *P. perfoliatum* have greater polarity and may be dominated by flavonoids. The bacteriostatic experimental studies on *P. perfoliatum* are all in vitro, and only the anti-bacterial activity of the crude extract was evaluated, but it is not clear which compound is the main anti-bacterial component. The bacteriostatic experiment of *P. perfoliatum* in vivo has not been reported.

### Anti-Viral

For a long time, *P. perfoliatum* has been widely used to treat diseases caused by viral infections such as herpes zoster (Xu et al., 2017). Modern pharmacological studies have found that ethanol extract, water extract, and n-butanol extract of *P. perfoliatum* have good antiviral activity, and it is found that the main active constituents are flavonoids and water-soluble phenolic acids. Zhang et al. (2010) tested the effect of *P. perfoliatum* extract against herpes simplex virus-1 (HSV-1) in vitro by MTT, and it was found that its ethanol extract and ethanol elution part of D101 macroporous resin had significant antiviral effect, which was similar to positive control drug acyclovir (ACV), and the virus inhibition rates were 78.10%, 75.2%, and 81.6%, respectively; taking rutin as the standard reference substance, the content of active extract was determined by aluminum nitrate complexation spectrophotometry, and the results showed that the content of flavonoids was more than 80%. Through further experimental studies, it was found that the flavonoids of *P. perfoliatum* had a strong inhibitory effect on the replication and intercellular transmission of HSV-1 virus (Fan et al., 2011), and could significantly prolong the survival time and improve the survival rate of mice with encephalitis induced by HSV-1 infection (Zhang et al., 2014). It is an effective method for the treatment of recurrent HSV-1 infection. However, it remains to be further studied on how the active components enter the cell and through what mechanism to block the virus infection and inhibit the replication of the virus.

The appropriate dose of *P. perfoliatum* water extract can effectively fight influenza virus and reduce lung inflammation, and it shows good antiviral effect by reducing the content of serum inflammatory factors TNF-α and IL-1, increasing the level of antibody IgA, IgG, IgG1b, and IgG2a, reducing lung index, and relieving lung inflammation in influenza mice (Wang et al., 2017); the mechanism is mainly through humoral immune response, and the antibodies in the serum can effectively neutralize the virus, activate the cascade of complements, and limit the replication and spread of pathogens (Lai et al., 2015). Quercetin-3-O-β-D-glucuronide, a flavonoid in *P. perfoliatum*, could significantly inhibit pulmonary edema induced by influenza A virus in mice, but the exact mechanism of action is not clear; the experimental results show that when the dose of quercetin-3-O-β-D-glucuronide is 6mg/ml, the inhibitory effect is stronger than that of the positive control drug ribavirin.

Wang (2009) reported that the water-soluble phenolic acids in PEE are the main active substances against hepatitis B virus (HBV), which can significantly inhibit the secretion of antigen (HBeAg) by HepG22.2.15 cells. The inhibition rate at non-toxic concentration can reach 74.02 ±11.43%, and the treatment index TI > 2.0, which is a low-toxic and efficient drug. Most of the anti-HBV active constituents are concentrated in n-butanol and water parts and contain common structural unit Gallic acyl groups. The more the number of Gallic acyl groups in the structure, the stronger the anti-HBV activity.

### Hepatoprotection and Anti-Liver Fibrosis

The liver is the main metabolic organ of the human body, and many factors, such as chemical drugs, pathogenic microorganisms, alcohol, and so on, can induce liver injury and cause liver cirrhosis, liver fibrosis, liver cancer, and other diseases (Xin and Qing, 2018). *P. perfoliatum* ethanol extract and total flavonoid extract have excellent hepatoprotective activity (He et al., 2019a). The ethanol extract of *P. perfoliatum* has a certain protective effect on acute immune liver injury induced by ConA in mice, and can effectively reduce the liver injury caused by concomitant inflammation. The mechanism may be related to the regulation of Kupffer cell function and reducing the synthesis and release of inflammatory mediator TNF- α (Wang, 2009). Total flavonoid extract of *P. perfoliatum* can reduce the activities of ALT, AST, TBA, TBIL, and LDH in serum, increase the activities of SOD and GSH in liver, reduce the content of lipid peroxide MDA, and it is suggested that the total flavonoids of *P. perfoliatum* can protect hepatocytes by inhibiting oxidative free radicals and improving the ability of antioxidation, and inhibit the lipid peroxidation induced by α-naphthalene isothiocyanate (ANIT) by reducing the content of lipid peroxidation product MDA in liver tissue (Cheng et al., 2018). In the study of the protective effect of *P. perfoliatum* on drug-induced liver injury induced by isoniazid and rifampicin in mice, it was found that under the intervention of total flavonoids of *P. perfoliatum*, the levels of TNF- α, IL-1β, and IL-6 in liver tissue of mice decreased, and the expression of Fas was significantly decreased by Western blotting assay, which had significant anti-drug-induced liver injury. The mechanism may be related to blocking Fas-mediated apoptosis pathway and inhibiting inflammatory response (Xi et al., 2017). The study of Xu et al. (2019) found that the extract of total flavonoids of *P. perfoliatum* could reduce the content of Hsp90, TNF- α, and IL-6 in the cells of mice with liver injury induced by carbon tetrachloride, increase the number of integrin β1, 5’- nucleotidase and positive antigen KI-67 cells in spleen, and reduce the pathological injury of liver; the mechanism may be through the inhibition of inflammatory stress reaction and apoptosis, inactivate Hsp90 in hepatocytes, and improve spleen immunity to protect liver. Total flavonoids of *P. perfoliatum* also have protective effects on acute alcoholic liver injury. It can improve antioxidation by scavenging lipid peroxides, reduce the content of inflammatory factors IL-4, IL-6, and TNF- α, and inhibit liver inflammation, while improving the immune capacity of the damaged body (Wei et al., 2016). In addition, the ethanol extract of *P. perfoliatum* not only alleviated the liver injury induced by carbon tetrachloride but also improved the structural damage of cardiomyocytes and glomeruli (He et al., 2019b).

In addition to the hepatoprotective effect, the total flavonoid extract of *P. perfoliatum* also showed significant anti-fibrosis activity (Cao, 2012) and had a good antagonistic effect on rat liver fibrosis induced by DMN (Cao et al., 2012; Guo and Shan, 2012). After the intervention of *P. perfoliatum* total flavonoid extract, the contents of serum HA, LN, PC III, IV-C, and other pathological indexes of liver fibrosis decreased, the degree of proliferation of hoof tissue in liver tissue decreased, the distribution of collagen decreased, and the degree of inflammation and fibrosis of liver tissue were greatly improved (Zhang et al., 2012); the mechanism is related to the fact that the flavonoid extract of *P. perfoliatum* can inhibit the expression of TGF- β1, prevent the activation of HSC into fibroblasts, inhibit the synthesis of extracellular matrix ECM, and promote its degradation (Gao et al., 2015).

In recent years, through the further study on the hepatoprotective mechanism of the total flavonoid extract of *P. perfoliatum*, Cao et al. (2017) have found that phosphorylated JAK2/STAT3 signal pathway can induce the expression of inflammatory cytokines in the process of liver fibrosis, and continuously secrete extracellular matrix ECM through positive feedback regulation of continuous activation of HSC; *P. perfoliatum* flavonoids can down-regulate the expression of α-SMA, TGF- β1, IL-1β, and IL-6, inhibit the activation of JAK2/STAT3 signal pathway, and improve liver pathological changes. In addition, the abnormal expression of TGF-β1 and JAK2/STAT3 pathway is consistent, and the relationship of mutual regulation between them has not been reported, which is worthy of further exploration. Gao et al.’s (2017) study found that *P. perfoliatum* flavonoids can significantly decrease the contents of TGF-β1, Notch-1, and α-SMA in liver tissue of rats with DMN-induced hepatic fibrosis, increased the expression of E-cadherin protein, inhibited the process of epithelial stroma transformation, and then reduced the secretion of extracellular matrix, preventing excessive deposition of fibrous hoof tissue. The mechanism is related to the regulation of TGF-β1/Notch signal pathway; some studies have shown that Notch-1 in Notch pathway is the effector gene of TGF- β1, and the flavonoids of *P. perfoliatum* may inhibit the EMT process induced by TGF-β 1 by blocking Notch signal pathway, so as to exert its anti-fibrosis effect.

### Antitussive and Expectorant

The syrup and decoction made of *P. perfoliatum* are clinically used in the treatment of pertussis, and the curative effect is remarkable (Zhan, 1996). It was found that the *P. perfoliatum* water extract inhibited the cough of ammonia-induced cough mice in a dose-dependent manner. The high dose group (0.2g/10g) significantly promoted the excretion of phenol red in the respiratory tract of mice in the phenol red expectorant experiment, and this shows that the higher dose of *P. perfoliatum* water extract has expectorant effect (Gu, 1996). Long and Li (2010) used SO_2_ to induce cough in mice and found that *P. perfoliatum* could prolong the cough latency of mice induced by SO_2_ stimulation, reduce the number of coughs, relieve cough significantly, and increase the sputum excretion of Wistar rats after drug modeling. Zhang et al. (2016) found that *P. perfoliatum* with honey can effectively prolong the latency of cough induced by concentrated ammonia water in mice, reduce the number of cough in mice, and the antitussive effect is stronger than crude plant.

### Anti-Tumor

The ethyl acetate extract from *P. perfoliatum* has a good inhibitory effect on tumor proliferation in vivo and in vitro (Tao and Zhang, 2013), and it can strongly inhibit the growth and proliferation of human gastric cancer SGC-7901 cells, colon cancer Colo320 cells, prostate cancer PC-3 cells, and acute myeloid leukemia HL60 cells in vitro (IC_50_ < 50ug/mL), but has a weak inhibitory effect on mouse melanoma B-16 cells (IC_50_ ≥ 91.63ug/mL). In vivo, the inhibition rate of *P. perfoliatum* ethyl acetate extract high dose group (200 mg/kg) on implanted sarcoma (S-180) in mice was 58.46%, and the inhibition rate of cyclophosphamide (CTX) in the positive control group was 52.31%.

Li et al. (2019) obtained an anti-tumor active component named PEC, through the separation of ethyl acetate extract from *P. perfoliatum*, and found that flavonoids were the main active components of PEC. PEC can effectively inhibit the proliferation of human cervical cancer cells, gastric cancer cells, prostate cancer cells, lung cancer cells, glioma cells, and pancreatic cancer cells in vitro. It also has obvious inhibitory effect on mouse hepatoma cell and sarcoma cell in vivo, and its inhibitory effect was dose-dependent. In addition, PEC can reduce the expression of VEGF in tumor tissue, reduce the density of microvascular system in tumor tissue, increase the activity of NK cells and cytotoxic lymphocytes (CTL), promote the proliferation of T lymphocytes and B lymphocytes, increase the activity of T lymphocytes, enhance the secretion of splenocyte IL-2, and raise the levels of IgG, IgG2a, and IgG2b in serum. It is suggested that its anti-tumor mechanism may be to inhibit the proliferation of tumor cells by improving immune ability, promoting tumor vascular necrosis, or hijacking tumor cells stagnated in G2 phase.

### Anti-Oxidation

The ethyl acetate and methanol extract isolated from *P. perfoliatum* can scavenge ABTS and DPPH free radicals in a dose-dependent manner. Cheng et al. (2020) obtained the polyphenol extract from *P. perfoliatum* by ultrasonic combined with Tween 80 extraction method, and found that it had strong antioxidant activity, at the same concentration, the scavenging rate and total reduction ability of polyphenol extract to DPPH free radicals was stronger than positive control drug Vitamin C, and the scavenging effect was positively correlated with the mass concentration. In addition, the extract of petroleum ether, ethyl acetate, and methanol from *P. perfoliatum* also had inhibitory effect on α-glucosidase activity (Xing et al., 2011), among which methanol extract had the strongest inhibitory activity, and the IC_50_ was 16.14 mg/L, which was much lower than positive control group (1081.27 mg/L).

## Quality Control

The current edition of the Pharmacopoeia of the People’s Republic of China (2020 edition) uses thin layer chromatography to qualitatively identify caffeic acid and high performance liquid chromatography (HPLC) to determine the content of quercetin, which is used to control the quality of *P. perfoliatum*; however, caffeic acid and quercetin exist widely in plants and their specificity is not strong, so it is necessary to use advanced technology and analytical methods to establish a scientific and reasonable quality evaluation system to provide a basis for the production and quality control of *P. perfoliatum*.

At present, the determination of index components in *P. perfoliatum* is still mostly determined by HPLC, but it is not limited to the determination of a single component, and multi-component determination has gradually become a hot spot. Cheng et al. (2013) used HPLC to determine quercetin and kaempferol in *P. perfoliatum*; Tong (2011) used HPLC to determine oleanolic acid and ursolic acid. Huang Jiayu used HPLC to determine ferulic acid and vanillic acid. Li et al. (2011) and Xu et al. (2009) determined the content of quercetin in different parts of *P. perfoliatum* by HPLC. Mao et al. (2010) determined the content of chlorogenic acid in *P. perfoliatum* by HPLC. Fan et al. (2014) established a HPLC for the simultaneous determination of esculetin, quercetin, quercetin-3-O-β-D-glucuronide, and quercetin-3-O-β-D-glucuronide-6″-methyl ester. Liu et al. (2010) determined the content of quercetin in stems and leaves of *P. perfoliatum* in different harvest periods by HPLC, it was found that the content of quercetin in leaves was significantly higher than that in stems, and August is the best harvest season. In addition, some advanced analytical techniques and determination methods have been gradually applied to the quality control of *P. perfoliatum*. Li et al. (2012) separated and determined ferulic acid, caffeic acid, and protocatechuic acid in *P. perfoliatum* by sweeping-MEKC. Jin et al. (2009) simultaneously determined quercetin, vanillic acid, ferulic acid, caffeic acid, and protocatechuic acid in *P. perfoliatum* by Electrophoretic-electrochemical method (CE-ED).

In recent years, fingerprint has been widely used in the quality control of traditional Chinese medicine. Tian et al. (2013) tested the 51 batches of *P. perfoliatum* samples collected from different locations or in different harvesting times in China by establishing the HPLC fingerprint determination method of *P. perfoliatum*, than analyzed the fingerprint obtained by chemometrics, similarity analysis, cluster analysis, and principal component analysis. Finally, the characteristic map is obtained. On the basis of the above, the pharmacodynamic similarities and differences of different batches of *P. perfoliatum* with statistical differences were analyzed. Finally, the researchers used mathematical statistical methods such as grey correlation degree method to statistically analyze the chemical information characterized by the chromatographic peaks of the chemical fingerprints of different batches of *P. perfoliatum* and the biological information characterized by the results of pharmacodynamics. Look for chemical substances that are closely related to pharmacodynamics. These studies have laid a good foundation for the identification of active components and quality control of *P. perfoliatum*.

## Conclusion and Prospect

*P. perfoliatum* is a kind of folk traditional Chinese medicine, which has a long history and definite curative effect in China. It is clinically effective in the treatment of herpes zoster, pertussis, acute bacillary dysentery, and gynecological inflammation. However, the research on *P. perfoliatum* is not comprehensive and in-depth in many aspects, and this paper discusses the research direction and focus of *P. perfoliatum* in the future from the following aspects:

① At present, 80 chemical constituents have been isolated from *P. perfoliatum*. Among them, flavonoids are the main active constituents, accounting for 32.5% of the isolated chemical constituents. The existing studies on the chemical constituents of *P. perfoliatum* are mostly focused on flavonoids, while the studies on other chemical constituents are less. For example, although the content of phenolic acids is much less than that of flavonoids, it still shows significant pharmacological activity, and according to the results of previous studies, phenolic acids may be the material basis of anti-inflammatory and antiviral effects. In addition, as the most important and abundant active components in *P. perfoliatum*, the research on flavonoids is not deep enough; study on the basic substances of pharmacology is still in the crude extract stage, and the specific active compounds have not been isolated and identified, which makes it difficult to establish the relationship between the chemical constituents and pharmacological activities, thus affecting further research in all aspects. Therefore, in the future, we should strengthen the basic research on the chemical constituents and pharmacological activities of *P. perfoliatum*.

② In recent years, the research found that *P. perfoliatum* total flavonoid extract has a good protective effect on liver tissue injury, and can improve and repair the damaged liver tissue structure, showing a good prospect of development and application in hepatoprotective and anti-hepatic fibrosis. However, many studies have reported that the pharmacological mechanisms of hepatoprotection and anti-hepatic fibrosis are not comprehensive and specific enough. Most of the studies on active constituents stay at crude extracts (mainly total flavonoid extracts), and there is a lack of in-depth study on monomer compounds. Therefore, it is necessary to screen and predict the action targets of active substances by means of systematic and network pharmacology, combined with modern research methods, and to clarify its specific pharmacological mechanism. At the same time, we further explore the biological activity and potential action mechanism of the extract of total flavonoids from *P. perfoliatum*.

③ In China, *P. perfoliatum* has made an outstanding contribution to the treatment of viral infectious diseases such as herpes zoster. The traditional Chinese medicine syrup made of *P. perfoliatum*, sucrose, and benzoic acid is effective in clinical application. According to literature reports, *P. perfoliatum* also has a significant effect on pertussis, an acute respiratory infectious disease caused by Bordetella pertussis. These applications show that *P. perfoliatum* has great potential in the treatment of respiratory diseases. However, these reports are limited to clinical observation reports, and there is a lack of research on active constituents and potential mechanism of action. Although most studies have summarized the active constituents and action mechanism of *P. perfoliatum*, they are not comprehensive and specific enough. Therefore, it is necessary to further strengthen the basic research on antiviral, antitussive, and expectorant.

④ *P. perfoliatum* has many traditional uses, but its over-extensive use highlights the deficiency of pharmacological activity research, and because traditional Chinese medicine has the characteristics of many ingredients and many action targets, it makes the research of pharmacological activity more difficult. In order to develop and utilize the resources, it is necessary to understand the pharmacological mechanism behind these uses, which also needs to further strengthen the existing pharmacological activity research. At the same time, the connection with traditional knowledge should be strengthened, and the importance of traditional clinical application in research and development should not be ignored, so as to better carry out the research on the pharmacology and efficacy of *P. perfoliatum* and find its specific range of drug use.

⑤ Although the quality standard of *P. perfoliatum* has been clearly stipulated in the current edition of the Pharmacopoeia of the People’s Republic of China (2020 edition), it still needs to be further improved because of its weak specificity. A large number of scientific studies related to the determination of index components and the construction of fingerprints have made good progress, but more efforts are still needed to incorporate these results into the legal quality standards. It is necessary to establish a more reasonable and effective quality evaluation system, improve the quality of *P. perfoliatum*, ensure the safety and effectiveness of clinical drug use, and provide a reference basis for better development and use of *P. perfoliatum* resources.

In a word, the *P. perfoliatum* has great development potential and research value, which is worthy of more comprehensive and in-depth study.

## Author Contributions

JL and XQ conceived and designed the review. SY, YX, SJ, CH, and ZL contributed to the collection of literature and relevant information. JL wrote this paper with help from YZ and GS. All authors contributed to the article and approved the submitted version.

## Funding

Thanks for funding from the studio of ZL, a national famous veteran expert in traditional Chinese medicine.

## Conflict of Interest

The authors declare that the research was conducted in the absence of any commercial or financial relationships that could be construed as a potential conflict of interest.
